# Risk of second primary malignancies in patients with follicular lymphoma: a population-based study in the Netherlands, 1989-2018

**DOI:** 10.1038/s41408-021-00574-5

**Published:** 2021-11-13

**Authors:** Manette A. W. Dinnessen, Otto Visser, Sanne H. Tonino, Eduardus F. M. Posthuma, Nicole M. A. Blijlevens, Marie José Kersten, Pieternella J. Lugtenburg, Avinash G. Dinmohamed

**Affiliations:** 1 Department of Research and Development, Netherlands Comprehensive Cancer Organisation (IKNL), Utrecht, The Netherlands; 2 Amsterdam UMC, University of Amsterdam, Department of Hematology, Cancer Center Amsterdam, LYMMCARE (Lymphoma and Myeloma Center Amsterdam), Amsterdam, The Netherlands; 3 Department of Registration, Netherlands Comprehensive Cancer Organisation (IKNL), Utrecht, The Netherlands; 4 Department of Internal Medical, Reinier de Graaf Gasthuis, Delft, The Netherlands; 5 Department of Hematology, Leiden University Medical Center, Leiden, The Netherlands; 6 Department of Hematology, Radboud University Medical Center, Nijmegen, The Netherlands; 7 Department of Hematology, Erasmus MC Cancer Institute, University Medical Center, Rotterdam, The Netherlands; 8 Amsterdam UMC, Vrije Universiteit Amsterdam, Department of Hematology, Cancer Center Amsterdam, Amsterdam, The Netherlands; 9 Department of Public Health, Erasmus University Medical Center, Rotterdam, The Netherlands

**Keywords:** Cancer epidemiology, Cancer epidemiology, Risk factors

The improved longevity of patients with follicular lymphoma (FL) is offset by an increased risk of a variety of second primary malignancies (SPMs) compared to the general population [1, 2]. Though scarce, population-based studies that encompass both the pre-and post-rituximab era reported an increased risk of Hodgkin lymphoma (HL) and acute myeloid leukemia (AML) and oropharyngeal cancer, gastric cancer, respiratory cancer, melanoma, and non-melanoma skin cancer, and urogenital cancer [1, 2]. The etiology of SPMs in FL might reflect late sequelae of treatment for FL or prior malignancies (e.g. immune dysfunction or DNA damage), or the effect of shared etiologic factors, environmental exposures, and genetic susceptibility, or some combination of these influences [1–4].

Awareness of SPMs in FL patients is essential to mitigate potential sequelae of SPMs that might counteract the comparatively favorable prognosis of FL. Therefore, to complement and extend the currently sparse literature on SPM development in FL, this nationwide population-based study aimed to assess the risk to develop SPMs in various subgroups of FL patients in the Netherlands diagnosed during a 30-year period.

We selected all patients diagnosed with FL grades 1-3B between 1989–2018 from the Netherlands Cancer Registry (NCR)—which covers >95% of all malignancies in the Netherlands [5]—using International Classification of Diseases for Oncology morphology codes as described elsewhere [6]. Patients diagnosed at autopsy (*n* = 22) were excluded. SPMs (excluding basal cell carcinoma of the skin) diagnosed between 1989–2018 were identified through cross-linkage with the NCR. To avert the overestimation of hematological SPMs, we excluded diffuse large B-cell lymphoma and HL since these lymphomas may have been misclassified as SPMs when they were actually transformations of FL. Synchronous malignancies diagnosed within six months from FL diagnosis (*n* = 248; 1.8% of the total FL population) were excluded to minimize detection bias since synchronous malignancies probably reflect incidental findings instead of SPMs.

Standardized incidence ratios (SIRs) for all SPMs combined and pre-defined subtypes of SPMs (Supplemental Table 1) were calculated by dividing the number of observed SPMs by the number of expected SPMs from the general population. The expected number of malignancies was obtained by multiplying sex-, age- (5-year intervals), and calendar year-specific incidence rates from the NCR by the accumulated person-years at risk. Person-years at risk was calculated from the date of FL diagnosis until SPM diagnosis, death, or end-of follow (December 31, 2018), whichever occurred first. Patients with multiple SPMs were counted only once in the analysis of all SPMs combined, and the time at risk ended on the date the first SPM was diagnosed. In the analyses of SPM subtypes, patients with multiple SPMs contributed data regarding all subtypes, regardless of whether an SPM was preceded by one at another site. In the case of multiple metachronous SPMs at the same site, only the first SPM was included in the analysis. We assigned 95% confidence intervals (CIs) for the SIRs by assuming a Poisson distribution for the number of observed SPMs and used the criteria of non-overlapping CIs to show statistically significant differences between subgroups. The absolute excess risk (AER) per 10 000 person-years was estimated as the expected number of SPMs subtracted by the observed number of SPMs, divided by the person-years at risk. SIRs and AERs were presented overall and according to age groups (18–60 and >60 years), sex, period of diagnosis (1989–2002 [pre-rituximab era] and 2003–2018 [post-rituximab era]), and years since diagnosis (0.5–10 and >10 years). We could not present SIRs and AERs according to FL treatment as the NCR does not standardly ascertain this information. The Privacy Review Board of the NCR approved the use of anonymous data for this study.

A total of 13 652 FL patients (median age, 62 years; 9% with a prior malignancy) were followed for a median of 5.5 years (interquartile range [IQR], 2.2–10.7). Baseline characteristics of these patients are presented in Table 1. During follow-up, 1 672 patients (12%) developed at least one SPM after a median follow-up of 5.1 years (IQR, 1.8–10.0 years) at a median age of 69 years (IQR, 62–76 years). Among these 1 672 patients, 699 (42%), 498 (30%), and 474 (28%) were diagnosed with an SPM after 0.5–5, 5–10, and 10–30 years post-diagnosis, respectively. The cumulative probability of SPMs was 8.9% (95% CI, 8.4–9.5%), 17.5% (95% CI, 16.7–18.5%), and 26.1 (95% CI, 34.2–38.1%) at 5, 10, and 20 years, respectively. Supplemental Fig. 1 and 2 depict the cumulative probability of all SPMs combined and SPM subtypes, respectively.

**Table 1:** Patient characteristics. Abbreviations: ASR, age-standardized incidence rate; IQR, interquartile range.

Characteristics	1989-2002		2003–2018		Total	
	No.	(%)	No.	(%)	No.	(%)
**Number of patients**	4760		8892		13652	
**ASR**	2.12		2.73		2.44	
**Sex**						
Male	2342	(49.2)	4571	(51.4)	6913	(50.6)
Female	2418	(50.8)	4321	(48.6)	6739	(49.4)
**Age, years**						
Median (IQR)	60	(49–70)	63	(54–71)	62	(52–71)
<18	12	(0.3)	10	(0.1)	22	(0.1)
18–60	2480	(52.1)	3776	(42.5)	6256	(45.8)
>60	2268	(47.7)	5106	(57.4)	7374	(54.0)
**Stage**						
I–II	1791	(37.6)	2944	(33.1)	4735	(34.7)
III–IV	2738	(57.5)	5797	(65.2)	8535	(62.5)
Unknown	231	(4.9)	151	(1.7)	382	(2.8)
**Follow-up, years**						
Median (IQR; range)	7.4	(2.7–17.1; 0.0–30.0)	4.9	(2.0–9.1; 0.0–16.0)	5.5	(2.2–10.7; 0.0–30.0)

Age-standardized incidence rates are age-adjusted to the European standard population and expressed per 100 000 person-years.

The overall risk of developing an SPM was statistically significantly elevated compared to the general population with a SIR of 1.42 (95% CI, 1.35–1.49) and an AER of 57.29 (Fig. 1). The overall excess risk of SPMs was comparable between the post- (SIR 1.33; 95% CI, 1.25–1.42; AER 49.17) and pre-rituximab era (SIR 1.53; 95% CI, 1.42–1.64; AER 65.88; Supplemental Fig. 3). We noted a higher SIR and AER for SPMs that were diagnosed beyond ten years post-diagnosis (SIR, 1.61; 95% CI, 1.47–1.76; AER, 94.28) compared to those diagnosed within 0.5–10 years post-diagnosis (SIR, 1.36; 95% CI, 1.28–1.43; AER, 46.78; Supplemental Fig. 4). The overall risk of developing SPMs beyond ten years post-diagnosis was comparable between the pre- (SIR, 1.33; 95% CI, 1.23–1.42) and post-rituximab era (SIR, 1.39; 95% CI, 1.10-1.67). Also, probably due to the lower baseline risk of developing malignancies among individuals 18-60 years compared to individuals >60 years, SIRs were higher among patients 18-60 years at diagnosis (SIR, 1.65; 95% CI, 1.53–1.77) compared to patients >60 years at diagnosis (SIR, 1.27; 95% CI, 1.19–1.36; Supplemental Fig. 5). Nevertheless, the AER was somewhat higher among patients >60 years (59.70) compared to patients 18–60 years (55.80; Supplemental Fig. 5). The elevated risk of SPMs was comparable between male (SIR 1.46; 95% CI, 1.37–1.56; AER 68.80) and female patients (SIR 1.37; 95% CI, 1.28–1.48; AER 46.23; Supplemental Fig. 6).

**Fig. 1:**
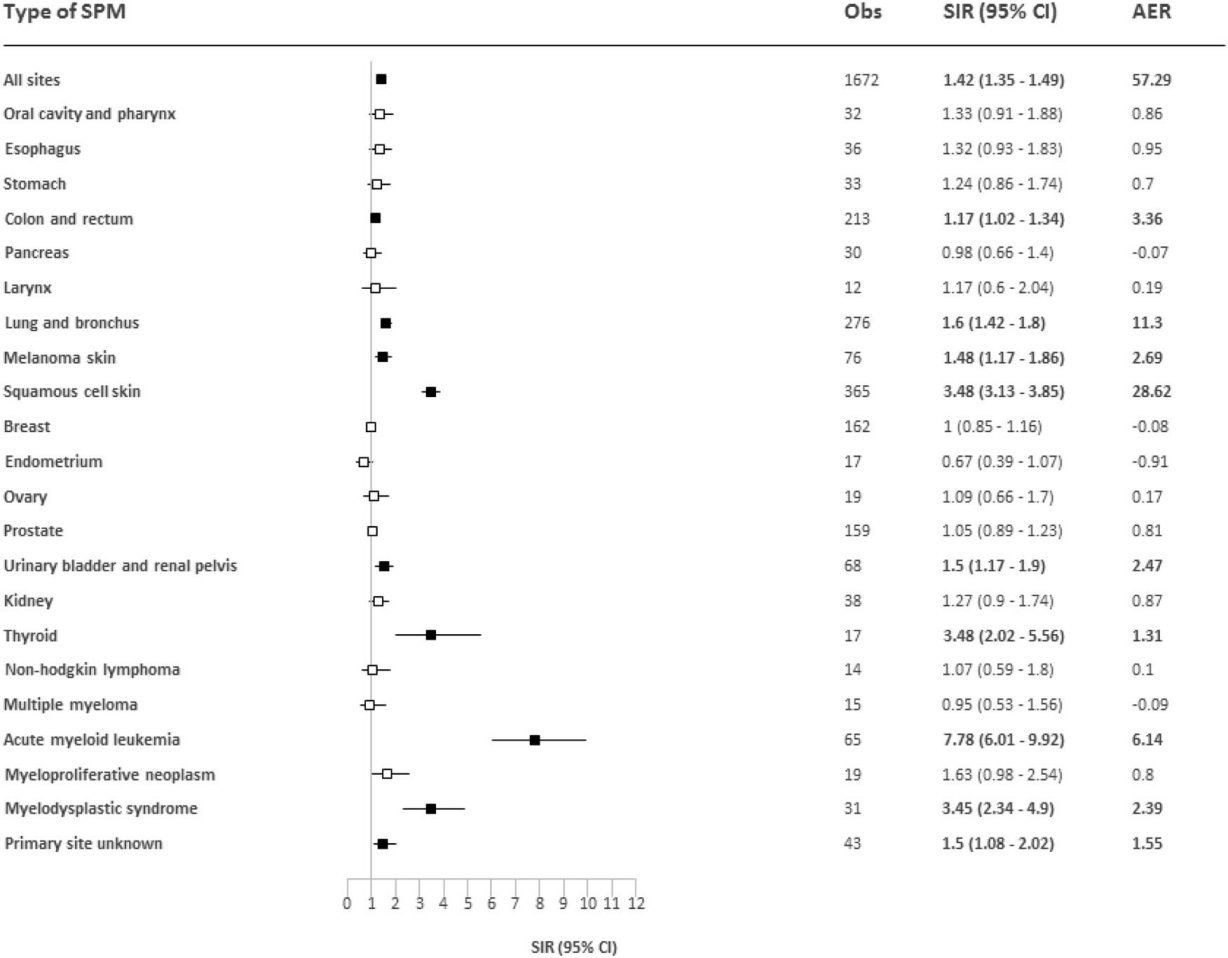
Risk of second primary malignancies (SPMs) compared to the general population among patients with follicular lymphoma. The tables present the observed number of SPMs (Obs), the standardized incidence ratios (SIRs) with 95% confidence intervals (CIs), and the absolute excess risk (AER) per 10 000 person-years. Statistically significant SIRs are presented in bold in the tables and as solid black boxes in the plots that visualize the SIRs with 95% CIs.

Squamous cell carcinomas of the skin contributed most to the AER across all subgroups, followed by lung and bronchus tumors and AML (Supplemental Figs. 3–6). Also, the spectrum of SPM subtypes was roughly comparable across most subgroups, except for the two age groups (Supplemental Figs. 3–6). Among patients aged 18–60 years, but not among patients aged >60 years, an elevated SPM risk was noted for non-Hodgkin lymphoma (NHL), myeloproliferative neoplasms (MPNs), and myelodysplastic syndromes (MDS), as well as solid tumors of the oral cavity and pharynx, stomach, colon and rectum, kidney, and primary site unknown (Supplemental Fig. 5).

In this nationwide population-based study, we observed that FL patients have a 42% greater risk of developing SPMs than the general population, both in the pre- and post-rituximab era. This finding corresponds with a population-based study from the USA in patients diagnosed during 1992-2011 [1] and a multicenter study among patients diagnosed during 1997–2016 in Spain and Finland [2]. These prior studies did not perform a formal comparison between the pre- and post-rituximab era. Our observations, with extended follow-up up to 2018, support the hypothesis that rituximab might not add to the already immunosuppressive and DNA damaging effects associated with alkylating agents and topoisomerase inhibitors used to manage FL [7, 8]. Indeed the SPM subtypes that are convincingly elevated among FL patients, are the subtypes that are believed to be associated with immune dysfunction and the use of chemotherapeutics (i.e. AML and MDS [2] and tumors of the lung and bronchus [9], skin [10, 11], urinary bladder, renal pelvis, and kidney [12]). This hypothesis is also strengthened by the observation that the time between FL diagnosis and SPM diagnosis was long enough to possibly be induced by immune dysfunction and the use of chemotherapeutics (Supplemental Fig. 1–3).

While most of our findings agree with the two above-mentioned population-based studies, some discrepancies require brief consideration. First, we could not objectify a lower than expected incidence of breast and pancreatic cancer shown in the study from the USA. Since there is no obvious biological explanation for these associations at this moment, they should be validated in forthcoming studies. Second, as opposed to the study from the USA, we demonstrated an elevated risk of thyroid cancer among FL patients. While an increased rate of incidental findings of thyroid cancer (or other cancers) among patients undergoing imaging studies is probable (i.e., surveillance bias), the association between FL and a heightened thyroid cancer risk warrants further attention since similar findings were observed among patients with other NHLs [13, 14]. Also, it is not common practice to perform imaging studies during routine follow-up after treatment or during a watch and wait approach in the Netherlands. A plausible biological explanation for this heightened risk could be the late effects of radiotherapy for FL management. Indeed, multiple studies have found that radiotherapy can induce thyroid cancer after a latency period of 5–10 years [15], congruent with our study findings (Supplemental Fig. 2).

The strength of our nationwide, population-based study is the use of comprehensive and complete data with 30 years of follow-up. Limitations include the possible misclassification of hematological SPMs and the lack of detailed clinical and treatment data throughout most of the registry. Also, due to the rarity of FL and the comparatively short follow-up period in the post-rituximab era, we did not have enough power to perform additional stratified analyses (e.g. SPM subtype analysis beyond ten years post-diagnosis in the post-rituximab era). Notwithstanding these limitations, our study findings bring to attention that FL patients have a greater risk of developing an SPM than the general population, also in the rituximab era. The excess risk of particular SPMs might be linked to immune dysfunction related to FL and its treatment and DNA damage related to FL treatment. Therefore, risk-based surveillance for SPMs remains vital in the survivorship care of FL patients to prevent or reduce the impact of SPMs.

## Supplementary information

Supplemental Material
