# Supplementary material for: Risk of second primary malignancies in patients with follicular lymphoma: a population-based study in the Netherlands, 1989-2018
Source: Blood Cancer J. 2021 Nov 13;11(11):179. doi: 10.1038/s41408-021-00574-5 (PMC8590687; doi:10.1038/s41408-021-00574-5)
Supplement: Supplementary file 1 — Supplemental Material [file 41408_2021_574_MOESM1_ESM.pdf]

ONLINE APPENDIX

Title

Risk of second primary malignancies in patients with follicular lymphoma: a population-based study in the Netherlands, 1989-2018

Running title

Second primary malignancies in follicular lymphoma

Authors and affiliations

Manette A.W. Dinnessen,[1,2] Otto Visser,[3] Sanne H. Tonino,[2] Eduardus F.M. Posthuma,[4,5] Nicole M.A. Blijlevens,[6] Marie José Kersten,[2] Pieternella J. Lugtenburg,[7] Avinash G. Dinmohamed[1,3,8,9]

[1]Department of Research and Development, Netherlands Comprehensive Cancer Organisation (IKNL), Utrecht, The Netherlands; [2]Amsterdam UMC, University of Amsterdam, Department of Hematology, Cancer Center Amsterdam, LYMMCARE (Lymphoma and Myeloma Center Amsterdam), Amsterdam, The Netherlands; [3]Department of Registration, Netherlands Comprehensive Cancer Organisation (IKNL), Utrecht, The Netherlands; [4]Department of Internal Medical, Reinier de Graaf Gasthuis, Delft, The Netherlands; [5]Department of Hematology, Leiden University Medical Center, Leiden, The Netherlands; [6]Department of Hematology, Radboud University Medical Center, Nijmegen, The Netherlands; [7]Department of Hematology, Erasmus MC Cancer Institute, University Medical Center, Rotterdam, The Netherlands; [8]Amsterdam UMC, Vrije Universiteit Amsterdam, Department of Hematology, Cancer Center Amsterdam, Amsterdam, The Netherlands; [9]Department of Public Health, Erasmus University Medical Center, Rotterdam, The Netherlands

Supplemental Table 1.

Supplemental Table 1. Definitions of subtypes of second primary malignancies with corresponding topography and morphology codes as per the International Classification of Diseases for Oncology.

SPM subtype	Included malignancies	Topography	Morphology (/3)
	Tongue carcinoma	C02	8000-8576, 8940-8941, 8980, 8982, 9110, 9990
	Gum carcinoma	C03	8000-8576, 8940-8941, 8980, 8982, 9110, 9990
	Floor of mouth carcinoma	C04	8000-8576, 8940-8941, 8980, 8982, 9110, 9990
	Palate carcinoma	C05.0, C05.8-9	8000-8576, 8940-8941, 8980, 8982, 9110, 9990
	Oral cavity, other/NOS	C00.3-5, C06	8000-8576, 8940-8941, 8980, 8982, 9110, 9990
**Oral cavity and pharynx**	Oropharynx carcinoma	C01, C05.1-2, C09, C10.0, C10.2-4, C10.8-9	8000--8576, 8940-8941, 8980, 8982, 9110, 9990
	Nasopharynx carcinoma	C11	8000-8576, 8940-8941, 8980, 8982, 9110, 9990
	Hypopharynx carcinoma	C12-13	8000-8576, 8940-8941, 8980, 8982, 9110, 9990
	Mouth and pharynx, other/NOS	C14.0, C14.2, C14.9	8000-8576, 8940-8941, 8980, 8982, 9110, 9990
	Parotid gland carcinoma	C07	8000-8576, 8940-8941, 8980, 8982, 9110, 9990
	Other salivary glands	C08	8000-8576, 8940-8941, 8980, 8982, 9110, 9990
**Esophagus**	Cervical oesphageal carcinoma	C15.0	8000-8149, 8154, 8158-8230, 8243-8246, 8250-8576, 8980, 8982, 9110, 9990
	oesophageal carcinoma (excl cervical)	C15.1-9	8000-8149, 8154, 8158-8230, 8243-8246, 8250-8576, 8980, 8982, 9110, 9990
**Stomach**	Invasive cardia carcinoma	C16.0	8000-8149, 8154, 8158-8230, 8243-8246, 8250-8576, 8980, 8982, 9110, 9990
	Invasive stomach carcinoma	C16.1-9	8000-8149, 8154, 8158-8230, 8243-8246, 8250-8576, 8980, 8982, 9110, 9990
	Colon carcinoma	C18.0, C18.2-9	8000-8231, 8243-8246, 8250-8576, 8980, 8982, 9110, 9990
	Appendix carcinoma	C18.1	8000-8231, 8243-8246, 8250-8576, 8980, 8982, 9110, 9990
**Colon and rectum**	Rectum carcinoma	C20	8000-8231, 8243-8246, 8250-8576, 8980, 8982, 9110, 9990
	Rectosigmoid carcinoma	C19	8000-8231, 8243-8246, 8250-8576, 8980, 8982, 9110, 9990
**Pancreas**	Exocrine pancreatic carcinoma	C25	8012, 8014-8040, 8045-8148, 8154, 8160-8231, 8243-8245, 8250-8573, 8575-8576, 8980, 8982
	Pancreatic carcinoma	C25.0-3, C25.5-9	8000-8011, 9990

SPM subtype	Included malignancies	Topography	Morphology (/3)
**Larynx**	Supraglottic carcinoma	C32.1, C10.1	8000-8576, 8940-8941, 8980, 8982, 9110, 9990
	Glottic carcinoma	C32.0	8000-8576, 8940-8941, 8980, 8982, 9110, 9990
	Subglottic carcinoma	C32.2	8000-8576, 8940-8941, 8980, 8982, 9110, 9990
	Other/unspecified laryngeal carcinoma	C32.8-9	8000-8576, 8940-8941, 8980, 8982, 9110, 9990
**Lung and bronchus**	Non small-cell lung carcinoma	C34	8010-8020, 8022-8035, 8046-8230, 8243-8246, 8250-8576, 8972, 8980-8982, 9110
	Small-cell lung carcinoma	C34	8002, 8021, 8041-8045
	Carcinoid of the lung	C34	8240-8242, 8248-8249
	Other/unspecified lung cancer	C34	8000-8001, 8003-8005, 9990, 8720-8790
	Pleuropulmonal blastoma	C34	8973
**Melanoma skin**	Melanoma of the skin and lip	C00.0, C00.1, C44	8720-8790
	Melanoma of the vulva	C51	8720-8790
	Melanoma of the penis	C60	8720-8790
	Melanoma of the scrotum	C63.2	8720-8790
	Melanoma of a primary unknown site	C80	8720-8790
**Squamous cell skin**	Squamous cell carcinoma of the eye lid	C44.1	8000-8012, 8014-8035, 8050-8084, 8560, 8575, 8980, 9990
	Squamous cell carcinoma other skin sites	C44.5-9, C63.2	8000-8012, 8014-8035, 8050-8084, 8560, 8575, 8980, 9990
	Squamous cell carcinoma head and nek	C44.0, C44.2-4	8000-8012, 8014-8035, 8050-8084, 8560, 8575, 8980, 9990
**Breast**	Invasive breast cancer carcinoma	C50	8000-8576, 8980, 8982, 8983, 9110, 9990
**Endometrium**	Endometrium carcinoma	C54-55	8000-8576, 8950-8951, 8980-8982, 9110, 9990

SPM category	Tumor subtype	Topography	Morphology (/3 unless otherwise indicated)
Ovary	Borderline ovarian carcinoma	C56, C48.1-2, C57	8000/1-8576/1, 9000/1-9015/1, 9110/1
	Borderline ovarian carcinoma	C56	8442/1, 8451/1, 8462/1, 8463/1, 8472/1, 8473/1,8460/2
	Non-epithelial ovarian tumors borderline	C56, C48.1-2, C57	8590/1-8670/1
	Non-invasive tuba carcinoma	C57.0	8000-8576, 8930-8934, 8950-8951, 8980, 8982, 9000-9015, 9110, 9990
	Epithelial ovarian carcinoma	C56	8000-8239, 8250-8441, 8450, 8452-8461, 8470-8471, 8474, 8480-8576, 8930-8934, 8950-8951, 8980, 8982, 9000-9015, 9110, 9990
	Extra-ovarian carcinoma	C48.1-2	8000, 8010-8149, 8154, 8158-8231, 8250-8576, 8950-8951, 8980, 8982, 9110
	Non-epithelial ovarian cancer	C56	8240-8249, 8590-8670, 9060-9105, 8800-8831, 8840-8850, 8852-8921, 8935, 8960-8973, 8990-8991, 9040-9044, 9120-9133, 9150-9261, 9364, 9380-9514, 9530-9581
Prostate	Prostate carcinoma	C61	8000-8576, 8980, 8982, 9110, 9990
Urinary bladder and renal pelvis	Muscle-invasive bladder carcinoma	C67.0-6, C67.8-9	8000-8576, 8980, 8982, 9110, 9990
	Carcinoma of the renal pelvis carcinoma	C65	8000-8576, 8980, 8982, 9110, 9990
Kidney	Kidney carcinoma	C64	8000-8576, 8980, 8982, 9110, 9990
Tyroid	Papillary and follicular carcinoma of the thyroid gland	C73	8010, 8046, 8140, 8201, 8260, 8290, 8310, 8330, 8331, 8332, 8335, 8337, 8339, 8340, 8341, 8342, 8343, 8344, 8350, 8430, 8450, 8481, 8504, 8560, 8570
	Medullary carcinoma of the thyroid gland	C73	8240, 8246, 8345-8347, 8510-8511
	Anaplastic carcinoma of the thyroid gland	C73	8012, 8020, 8021, 8022, 8030, 8031, 8032, 8033, 8035, 8041, 8980
	Squamous cell carcinoma of the thyroid gland	C73	8051-8084
	Other/unspecified carcinoma of the thyroid gland	C73	8000-8005, 9990, 8588, 8589

SPM category	Tumor subtype	Topography	Morphology (/3)
	Lymphoplasmacytic lymphoma / Waldenström macroglobulinemia	all	9761
	Indolent NHL, other/NOS	all	9689, 9711, 9764
**Non-hodgkin lymphoma**	Hairy cell' leukaemia	all	9940, 9941
	B-PLL	all	9825, 9832, 9833, 9631
	Mantle cell lymphoma	all	9673, 9621, 9672
	Burkitt lymphoma / leukaemia	all	9687, 9826
**Multipel myeloma**	Multiple myeloma	all	9730, 9732
	AML with specific cytogenetic abnormalities	all	9865-9866, 9869, 9871, 9877-9879, 9896-9897, 9911, 9912
	AML with myelodysplasia-related changes	all	9895, 9984
	Therapy-related myeloid neoplasms	all	9920, 9987
**Acute myeloid leukemia**	AML, other/NOS	all	9840-9841, 9861, 9864, 9867, 9870, 9872-9874, 9890-9891, 9910, 9931-9932
	Myeloid sarcoma	all	9930
	Myeloid leukaemia associated with Down syndrome	all	9898
	Blastic plasmacytoid dendritic cell neoplasm	all	9727
	Chronic myeloid leukaemia	all	9863, 9875
	Mast cell tumors	all	9740-9742, 9900
**Myeloproliferative neoplasms**	Myelofibrosis	all	9961
	Myeloid and lymphoid neoplasms with eosinophilia and abnormalities of PDGFRA, PDGFRB or FGFR1	all	9965-9967, 9963-9964, 9968, 9880
	Myeloproliferative neoplasms, other/NOS	all	9842, 9950, 9960, 9962
**Myelodysplastic syndrome**	Myelodysplastic syndrome	all	9980, 9982-9983, 9985-9986, 9989, 9991-9993
**Primary site unknown**	Other/unspecified sites	C76, C39	8000-8576, 8980, 8982, 9110, 9990
	Primary site unknown	C80	8000-8148, 8154, 8160-8231, 8243-8246, 8250-8576, 8980-8982, 9110, 9990

Supplemental Figure 1.

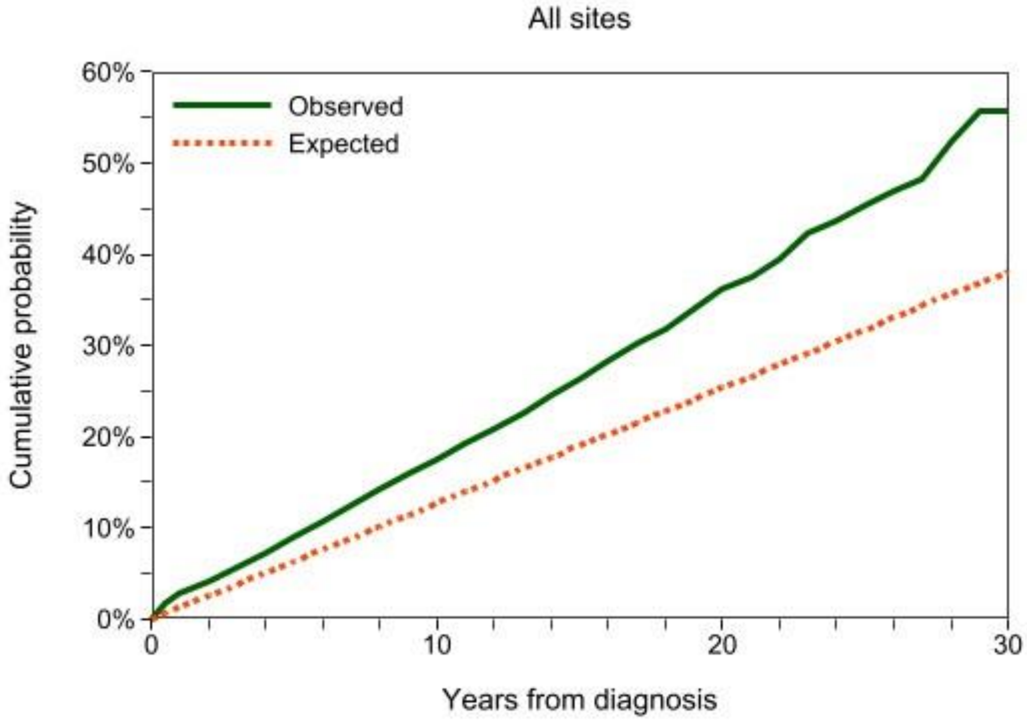

Supplemental Figure 1. Cumulative probability of a second primary malignancy (one minus Kaplan-Meier estimate) among patients with follicular lymphoma (observed) and the general Dutch population (expected).

Supplemental Figure 2

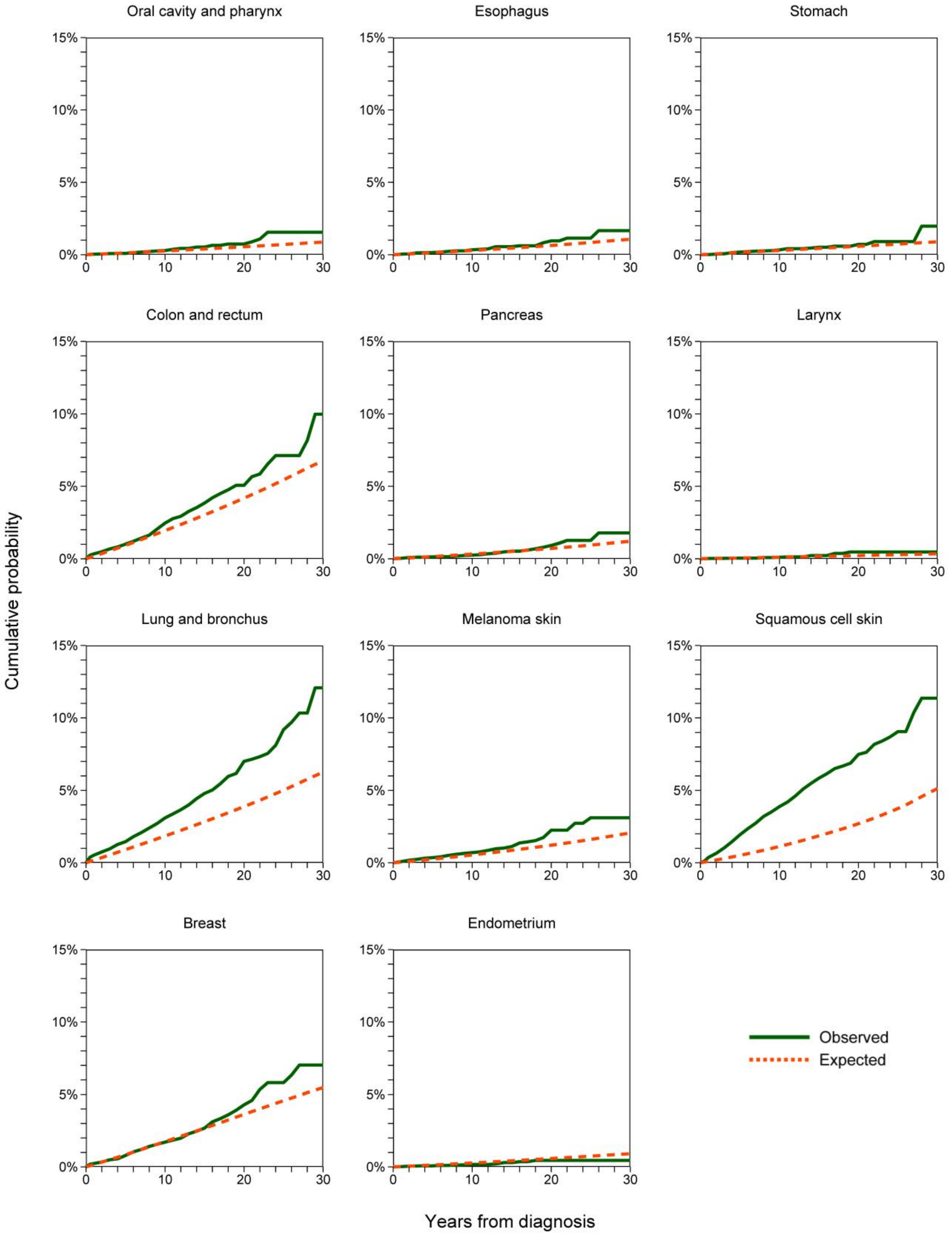

Supplemental Figure 2 (continued)

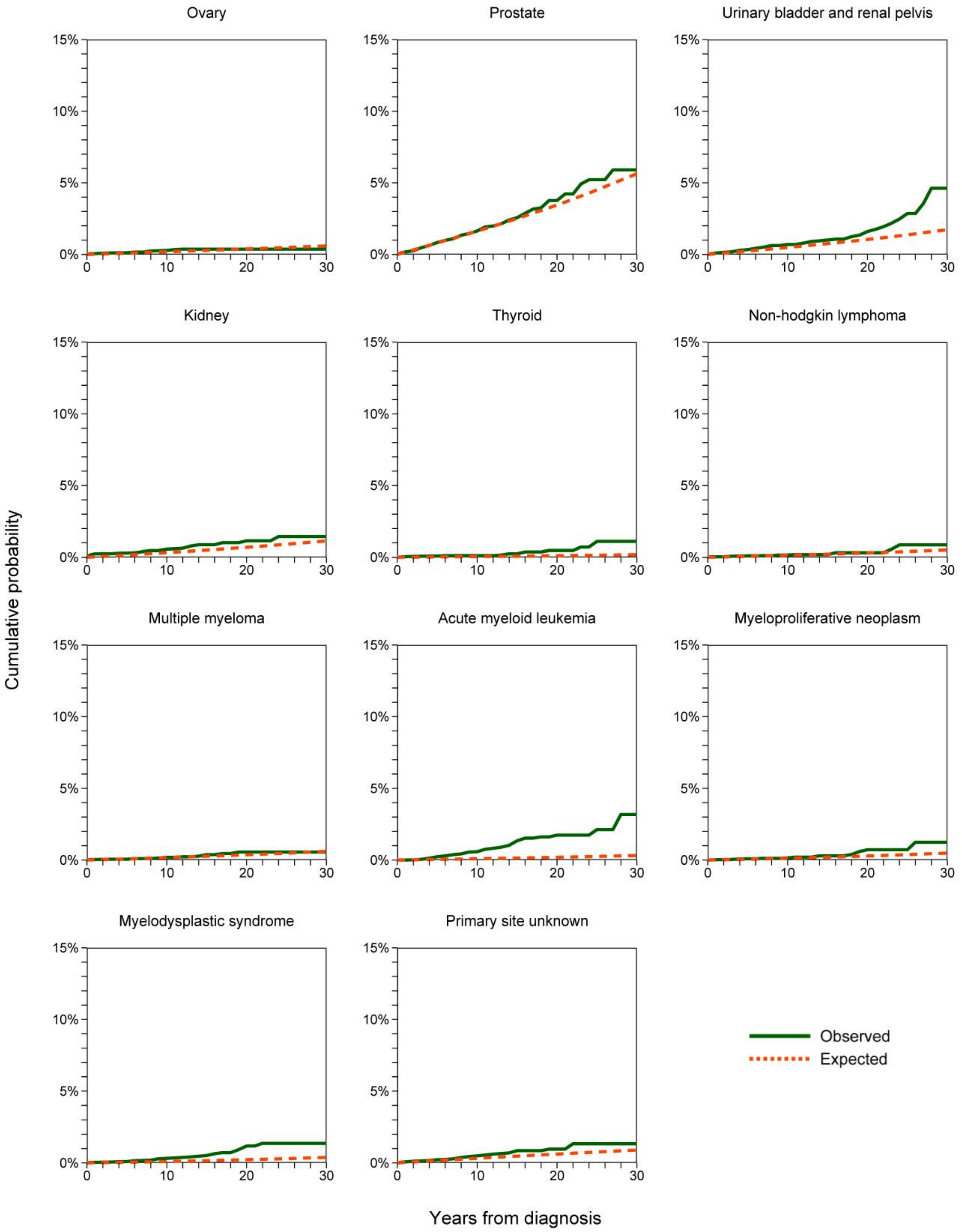

Supplemental Figure 2. Cumulative probability of subtypes of second primary malignancies (one minus Kaplain-Meier estimate) among patients with follicular lymphoma (observed) and the general Dutch population (expected).

Supplemental Figure 3.

Type of SPM	1989-2002 Obs	1989-2002 SIR (95% CI)	1989-2002 AER	2003-2018 Obs	2003-2018 SIR (95% CI)	2003-2018 AER
All sites	799	**1.53 (1.42 - 1.64)**	65.88	873	**1.33 (1.25 - 1.42)**	49.17
Oral cavity and pharynx	18	1.66 (0.98 - 2.62)	1.59	14	1.06 (0.58 - 1.78)	0.17
Esophagus	16	1.38 (0.79 - 2.25)	0.98	20	1.28 (0.78 - 1.97)	0.92
Stomach	21	1.46 (0.9 - 2.23)	1.47	12	0.98 (0.51 - 1.72)	-0.04
Colon and rectum	99	1.21 (0.98 - 1.47)	3.86	114	1.13 (0.94 - 1.36)	2.88
Pancreas	12	0.88 (0.45 - 1.53)	-0.38	18	1.06 (0.63 - 1.67)	0.21
Larynx	7	1.36 (0.55 - 2.8)	0.41	5	0.98 (0.32 - 2.28)	-0.02
Lung and bronchus	137	**1.72 (1.44 - 2.03)**	12.78	139	**1.5 (1.26 - 1.77)**	9.89
Melanoma skin	33	**1.67 (1.15 - 2.35)**	2.95	43	1.36 (0.99 - 1.84)	2.44
Squamous cell skin	150	**3.82 (3.24 - 4.49)**	24.93	215	**3.27 (2.85 - 3.74)**	32.14
Breast	88	1.15 (0.92 - 1.42)	2.57	74	0.86 (0.67 - 1.08)	-2.6
Endometrium	8	0.66 (0.28 - 1.29)	-0.93	9	0.68 (0.31 - 1.29)	-0.9
Ovary	10	1.11 (0.53 - 2.05)	0.23	9	1.07 (0.49 - 2.02)	0.12
Prostate	79	1.19 (0.95 - 1.49)	2.87	80	0.94 (0.74 - 1.17)	-1.16
Urinary bladder and renal pelvis	38	**1.8 (1.27 - 2.47)**	3.75	30	1.24 (0.84 - 1.77)	1.24
Kidney	20	1.5 (0.92 - 2.32)	1.49	18	1.08 (0.64 - 1.7)	0.28
Thyroid	8	**3.83 (1.65 - 7.54)**	1.31	9	**3.21 (1.47 - 6.1)**	1.31
Non-hodgkin lymphoma	6	1.02 (0.37 - 2.22)	0.02	8	1.11 (0.48 - 2.19)	0.17
Multiple myeloma	10	1.39 (0.67 - 2.56)	0.62	5	0.58 (0.19 - 1.35)	-0.78
Acute myeloid leukemia	36	**9.28 (6.5 - 12.85)**	7.12	29	**6.49 (4.35 - 9.32)**	5.2
Myeloproliferative neoplasm	7	1.62 (0.65 - 3.33)	0.59	12	1.64 (0.85 - 2.86)	0.99
Myelodysplastic syndrome	20	**5.73 (3.5 - 8.84)**	3.66	11	**2 (1 - 3.58)**	1.17
Primary site unknown	28	1.63 (1.09 - 2.36)	2.41	15	1.3 (0.73 - 2.14)	0.73

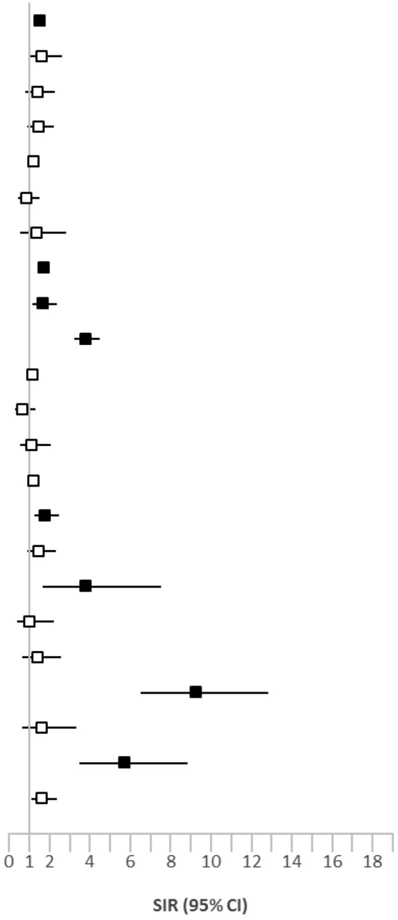

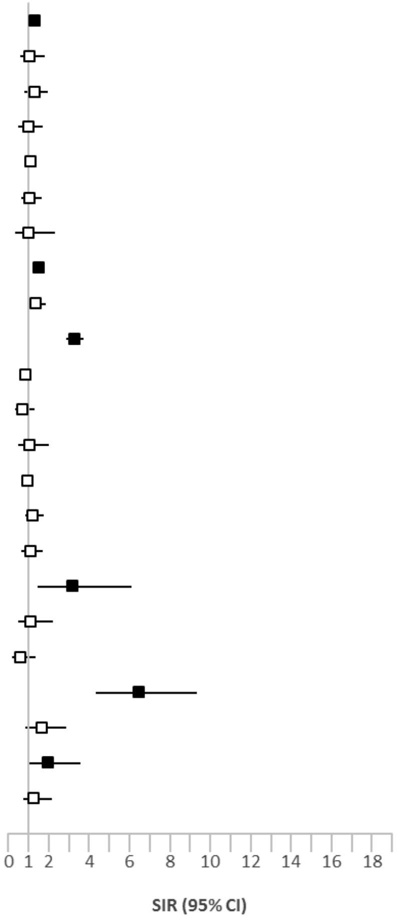

Supplemental Figure 3. Risk of second primary malignancies (SPMs) compared to the general population among patients with follicular lymphoma according to year of diagnosis. The tables present the observed number of SPMs (Obs), the standardized incidence ratios (SIRs) with 95% confidence intervals (CIs), and the absolute excess risk (AER) per 10,000 person-years. Statistically significant SIRs are presented in bold in the tables and as solid black boxes in the plots that visualize the SIRs with 95% CIs.

Supplemental Figure 4.

Type of SPM	0.5-10 years Obs	0.5-10 years SIR (95% CI)	0.5-10 years AER	>10 years Obs	>10 years SIR (95% CI)	>10 years AER
All sites	1198	**1.36 (1.28 - 1.43)**	46.78	474	**1.61 (1.47 - 1.76)**	94.28
Oral cavity and pharynx	18	1.01 (0.6 - 1.6)	0.03	14	**2.23 (1.22 - 3.74)**	3.56
Esophagus	24	1.22 (0.78 - 1.81)	0.61	12	1.6 (0.83 - 2.79)	2.07
Stomach	24	1.18 (0.76 - 1.76)	0.53	9	1.43 (0.66 - 2.72)	1.25
Colon and rectum	148	1.11 (0.94 - 1.3)	2.1	65	**1.33 (1.02 - 1.69)**	7.5
Pancreas	16	0.72 (0.41 - 1.17)	-0.89	14	1.66 (0.91 - 2.79)	2.56
Larynx	6	0.77 (0.28 - 1.68)	-0.25	6	2.43 (0.89 - 5.3)	1.63
Lung and bronchus	195	**1.53 (1.32 - 1.76)**	9.57	81	**1.82 (1.45 - 2.26)**	16.96
Melanoma skin	50	**1.36 (1.01 - 1.79)**	1.87	26	**1.8 (1.18 - 2.64)**	5.37
Squamous cell skin	283	**3.91 (3.46 - 4.39)**	30.2	82	**2.52 (2.01 - 3.13)**	23.4
Breast	108	0.89 (0.73 - 1.07)	-1.99	54	1.32 (0.99 - 1.73)	6.21
Endometrium	12	0.64 (0.33 - 1.12)	-0.96	5	0.75 (0.24 - 1.75)	-0.77
Ovary	16	1.22 (0.7 - 1.98)	0.41	3	0.7 (0.14 - 2.05)	-0.59
Prostate	111	0.99 (0.81 - 1.19)	-0.15	48	1.22 (0.9 - 1.61)	3.98
Urinary bladder and renal pelvis	47	**1.41 (1.04 - 1.88)**	1.95	21	**1.75 (1.08 - 2.68)**	4.16
Kidney	26	1.18 (0.77 - 1.73)	0.57	12	1.5 (0.78 - 2.62)	1.85
Thyroid	9	**2.5 (1.14 - 4.74)**	0.76	8	**6.22 (2.68 - 12.25)**	3.09
Non-hodgkin lymphoma	9	0.94 (0.43 - 1.78)	-0.08	5	1.43 (0.47 - 3.34)	0.7
Multiple myeloma	9	0.78 (0.35 - 1.47)	-0.37	6	1.41 (0.52 - 3.08)	0.81
Acute myeloid leukemia	39	**6.34 (4.51 - 8.67)**	4.65	26	**11.82 (7.72 - 17.33)**	10.97
Myeloproliferative neoplasm	10	1.22 (0.59 - 2.24)	0.26	9	**2.6 (1.19 - 4.93)**	2.55
Myelodysplastic syndrome	18	**2.88 (1.71 - 4.56)**	1.67	13	**4.75 (2.53 - 8.11)**	4.73
Primary site unknown	31	1.39 (0.95 - 1.98)	1.24	12	1.85 (0.96 - 3.24)	2.54

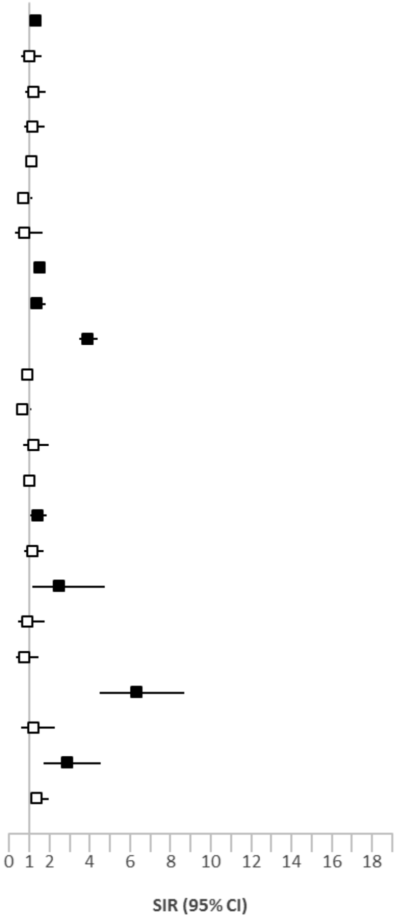

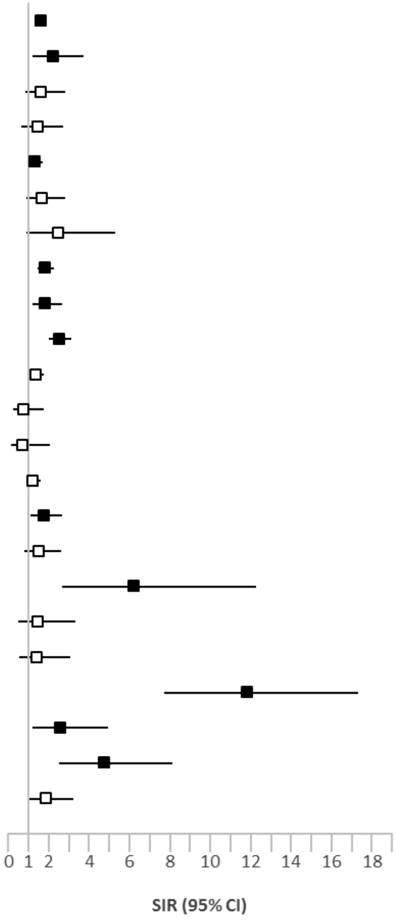

Supplemental Figure 4. Risk of second primary malignancies (SPMs) compared to the general population among patients with follicular lymphoma according to follow-up duration. The tables present the observed number of SPMs (Obs), the standardized incidence ratios (SIRs) with 95% confidence intervals (CIs), and the absolute excess risk (AER) per 10,000 person-years. Statistically significant SIRs are presented in bold in the tables and as solid black boxes in the plots that visualize the SIRs with 95% CIs.

Supplemental Figure 5.

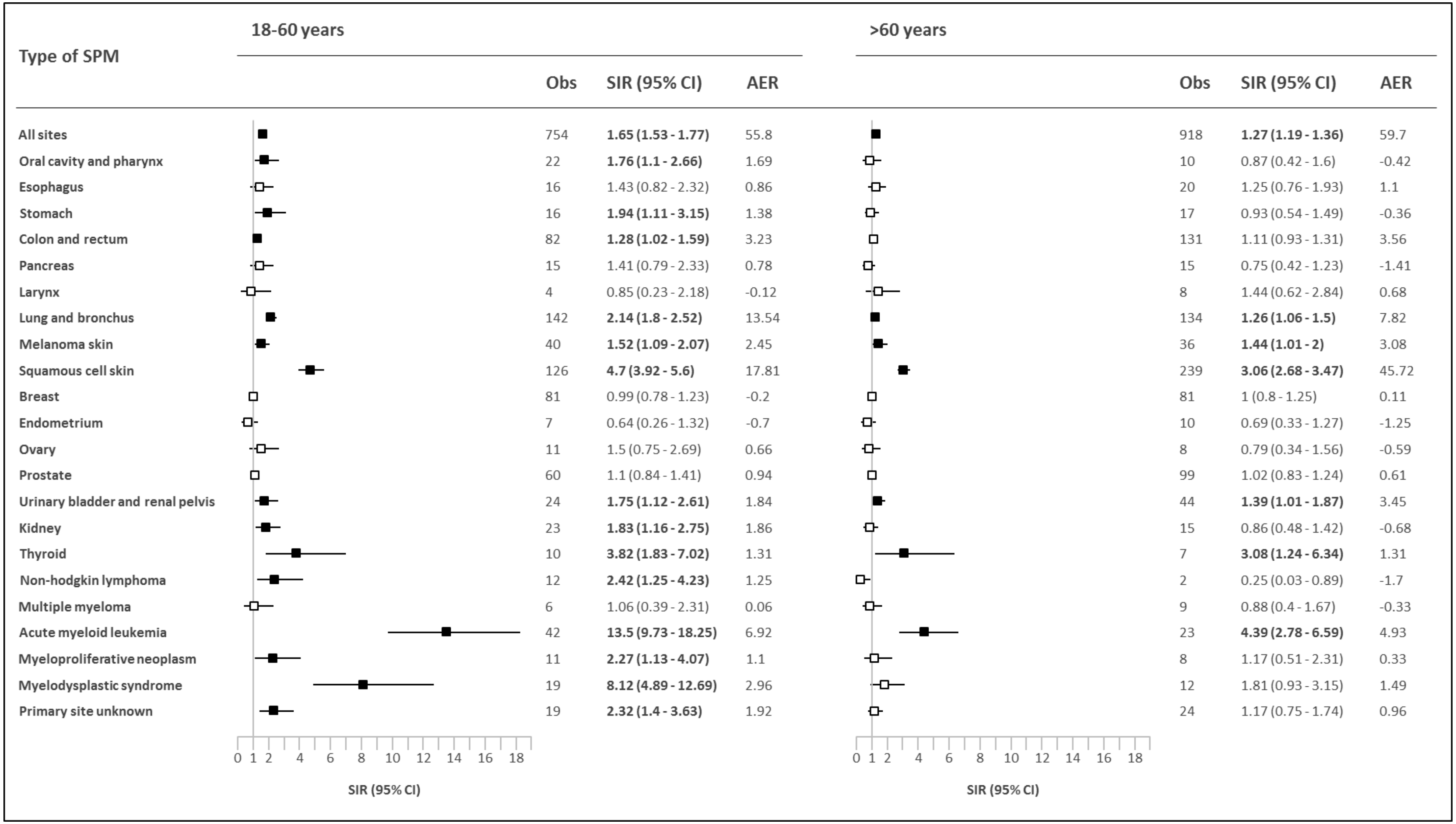

Type of SPM	18-60 years Obs	18-60 years SIR (95% CI)	18-60 years AER	>60 years Obs	>60 years SIR (95% CI)	>60 years AER
All sites	754	**1.65 (1.53 - 1.77)**	55.8	918	**1.27 (1.19 - 1.36)**	59.7
Oral cavity and pharynx	22	**1.76 (1.1 - 2.66)**	1.69	10	0.87 (0.42 - 1.6)	-0.42
Esophagus	16	1.43 (0.82 - 2.32)	0.86	20	1.25 (0.76 - 1.93)	1.1
Stomach	16	**1.94 (1.11 - 3.15)**	1.38	17	0.93 (0.54 - 1.49)	-0.36
Colon and rectum	82	**1.28 (1.02 - 1.59)**	3.23	131	1.11 (0.93 - 1.31)	3.56
Pancreas	15	1.41 (0.79 - 2.33)	0.78	15	0.75 (0.42 - 1.23)	-1.41
Larynx	4	0.85 (0.23 - 2.18)	-0.12	8	1.44 (0.62 - 2.84)	0.68
Lung and bronchus	142	**2.14 (1.8 - 2.52)**	13.54	134	**1.26 (1.06 - 1.5)**	7.82
Melanoma skin	40	**1.52 (1.09 - 2.07)**	2.45	36	**1.44 (1.01 - 2)**	3.08
Squamous cell skin	126	**4.7 (3.92 - 5.6)**	17.81	239	**3.06 (2.68 - 3.47)**	45.72
Breast	81	0.99 (0.78 - 1.23)	-0.2	81	1 (0.8 - 1.25)	0.11
Endometrium	7	0.64 (0.26 - 1.32)	-0.7	10	0.69 (0.33 - 1.27)	-1.25
Ovary	11	1.5 (0.75 - 2.69)	0.66	8	0.79 (0.34 - 1.56)	-0.59
Prostate	60	1.1 (0.84 - 1.41)	0.94	99	1.02 (0.83 - 1.24)	0.61
Urinary bladder and renal pelvis	24	**1.75 (1.12 - 2.61)**	1.84	44	**1.39 (1.01 - 1.87)**	3.45
Kidney	23	**1.83 (1.16 - 2.75)**	1.86	15	0.86 (0.48 - 1.42)	-0.68
Thyroid	10	**3.82 (1.83 - 7.02)**	1.31	7	**3.08 (1.24 - 6.34)**	1.31
Non-hodgkin lymphoma	12	**2.42 (1.25 - 4.23)**	1.25	2	**0.25 (0.03 - 0.89)**	-1.7
Multiple myeloma	6	1.06 (0.39 - 2.31)	0.06	9	0.88 (0.4 - 1.67)	-0.33
Acute myeloid leukemia	42	**13.5 (9.73 - 18.25)**	6.92	23	**4.39 (2.78 - 6.59)**	4.93
Myeloproliferative neoplasm	11	**2.27 (1.13 - 4.07)**	1.1	8	1.17 (0.51 - 2.31)	0.33
Myelodysplastic syndrome	19	**8.12 (4.89 - 12.69)**	2.96	12	1.81 (0.93 - 3.15)	1.49
Primary site unknown	19	**2.32 (1.4 - 3.63)**	1.92	24	1.17 (0.75 - 1.74)	0.96

Supplemental Figure 5. Risk of second primary malignancies (SPMs) compared to the general population among patients with follicular lymphoma according to age at diagnosis. The tables present the observed number of SPMs (Obs), the standardized incidence ratios (SIRs) with 95% confidence intervals (CIs), and the absolute excess risk (AER) per 10,000 person-years. Statistically significant SIRs are presented in bold in the tables and as solid black boxes in the plots that visualize the SIRs with 95% CIs.

Supplemental Figure 6.

Type of SPM	Male Obs	Male SIR (95% CI)	Male AER	Female Obs	Female SIR (95% CI)	Female AER
All sites	925	**1.46 (1.37 - 1.56)**	68.8	747	**1.37 (1.28 - 1.48)**	46.23
Oral cavity and pharynx	18	1.2 (0.71 - 1.9)	0.67	14	1.54 (0.84 - 2.59)	1.05
Esophagus	26	1.31 (0.85 - 1.92)	1.35	10	1.37 (0.66 - 2.51)	0.57
Stomach	22	1.26 (0.79 - 1.91)	1.01	11	1.2 (0.6 - 2.15)	0.39
Colon and rectum	109	1.08 (0.89 - 1.31)	1.85	104	**1.27 (1.04 - 1.54)**	4.82
Pancreas	15	0.97 (0.54 - 1.6)	-0.1	15	0.99 (0.55 - 1.63)	-0.05
Larynx	9	1.06 (0.48 - 2)	0.11	3	1.72 (0.36 - 5.04)	0.27
Lung and bronchus	155	**1.4 (1.19 - 1.64)**	9.89	121	**1.95 (1.62 - 2.33)**	12.66
Melanoma skin	41	**1.56 (1.12 - 2.12)**	3.25	35	**1.4 (0.98 - 1.95)**	2.15
Squamous cell skin	242	**4.08 (3.58 - 4.62)**	41.09	123	**2.7 (2.24 - 3.22)**	16.67
Breast	3	2.34 (0.48 - 6.84)	0.38	159	0.98 (0.84 - 1.15)	-0.54
Endometrium	-	-	-	17	0.67 (0.39 - 1.07)	-1.8
Ovary	-	-	-	19	1.09 (0.66 - 1.7)	0.34
Prostate	159	1.05 (0.89 - 1.23)	1.66	-	-	-
Urinary bladder and renal pelvis	49	**1.44 (1.07 - 1.9)**	3.31	19	**1.69 (1.02 - 2.64)**	1.65
Kidney	25	1.33 (0.86 - 1.96)	1.37	13	1.16 (0.62 - 1.99)	0.39
Thyroid	8	**4.83 (2.09 - 9.53)**	1.4	9	**2.78 (1.27 - 5.28)**	1.23
Non-hodgkin lymphoma	9	1.14 (0.52 - 2.16)	0.24	5	0.97 (0.31 - 2.25)	-0.04
Multiple myeloma	9	1.02 (0.47 - 1.93)	0.03	6	0.86 (0.31 - 1.86)	-0.21
Acute myeloid leukemia	34	**7.17 (4.96 - 10.01)**	6.45	31	**8.6 (5.84 - 12.21)**	5.84
Myeloproliferative neoplasm	10	1.73 (0.83 - 3.18)	0.93	9	1.53 (0.7 - 2.91)	0.67
Myelodysplastic syndrome	21	**3.75 (2.32 - 5.73)**	3.4	10	**2.95 (1.42 - 5.43)**	1.41
Primary site unknown	17	1.14 (0.66 - 1.83)	0.46	26	**1.88 (1.23 - 2.76)**	2.6

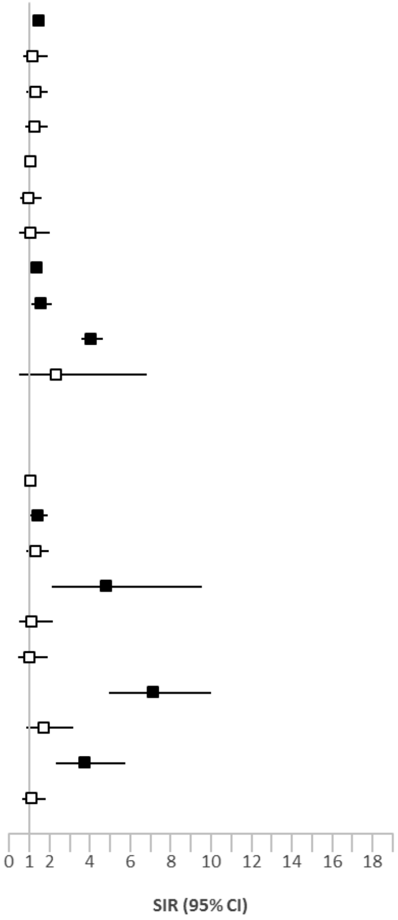

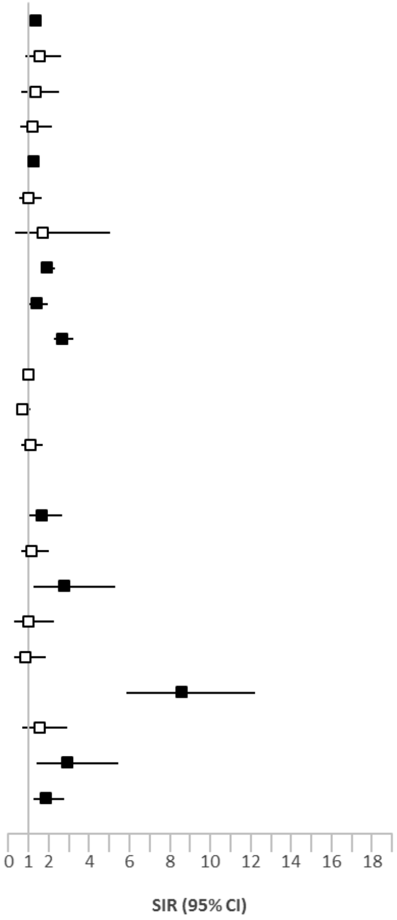

Supplemental Figure 6. Risk of second primary malignancies (SPMs) compared to the general population among patients with follicular lymphoma according to sex. The tables present the observed number of SPMs (Obs), the standardized incidence ratios (SIRs) with 95% confidence intervals (CIs), and the absolute excess risk (AER) per 10,000 person-years. Statistically significant SIRs are presented in bold in the tables and as solid black boxes in the plots that visualize the SIRs with 95% CIs.
